# Monitoring safety and effectiveness of Tafamidis in transthyretin amyloidosis in Italy: a 3-year longitudinal multicenter study in a non-endemic area

**DOI:** 10.1186/1750-1172-10-S1-P6

**Published:** 2015-11-02

**Authors:** Andrea Cortese, Giuseppe Vita, Laura Obici, Marco Sabatelli, Gian Maria Fabrizi, Angelo Schenone, Giampaolo Merlini, Davide Pareyson

**Affiliations:** 1 C. Mondino National Neurological Institute, General Neurology, 27100, Pavia, Italy; 2 University of Messina and NEMO SUD Center for Neuromuscular Disorders, Fondazione Aurora Onlus, Department of Neurosciences, 98121, Messina, Italy; 3 Amyloidosis Research and Treatment Center, Department of Molecular Medicine, Fondazione Istituto Di Ricovero e Cura a Carattere Scientifico Policlinico San Matteo and University of Pavia, 27100, Pavia, Italy; 4 Institute of Neurology, Catholic University of Sacred Heart, Department of Geriatrics, Neurosciences and Orthopedics, 00118, Roma, Italy; 5 University of Verona, Department of Neurological, Neuropsychological, Morphological and Motor Sciences, 37010, Verona, Italy; 6 University Federico II of Naples, Department of Neurosciences, Reproductive and Odontostomatological Sciences, 80121, Napoli, Italy; 7 IRCCS Foundation, C. Besta Neurological Institute, Department of Neurolog, 20124, Milano, Italy

## Background

Tafamidis is a transthyretin (TTR) stabilizer able to prevent mutated TTR tetramer dissociation into amyloidogenic monomers. There have been a few encouraging studies on safety and long-term efficacy of Tafamidis in early-onset Val30Met TTR-familial amyloid polyneuropathy (TTR-FAP) patients. However, less is known about its efficacy in later stages of the disease and in non-Val30Met mutations.

## Methods

Multi-center observational study on symptomatic TTR-FAP patients prescribed to receive tafamidis. We followed up patients according to a standardized protocol including general medical, cardiological and neurological assessments at baseline and every 6 months up to 3 years.

## Results

61 (42 males) patients were recruited. Only 28% of enrolled subjects had the common Val30Met mutation, mean age of onset was remarkably late (59 years) and 18% was in an advanced disease stage at study entry. Tafamidis proved safe and well-tolerated. One third of patients did not show significant progression along 36 months, independently from mutation type and disease stage. Neurological function worsened particularly in the first 6 months but slowed significantly thereafter. Fifteen percent of patients showed cardiac disease progression and 30% new onset of cardiomyopathy. A higher mBMI at baseline was associated with better preservation on neurological function.

## Conclusions

Neuropathy and cardiomyopathy progressed in a significant proportion of patients despite treatment. However, the worsening of neurological function slowed after the first 6 months and also subjects with more advanced neuropathy, as well as patients with non-Val30Met mutation, benefited from Tafamidis treatment. Body weight preservation is an important favorable prognostic factor.

